# Lung Nodule Image Classification Based on Local Difference Pattern and Combined Classifier

**DOI:** 10.1155/2016/1091279

**Published:** 2016-12-07

**Authors:** Keming Mao, Zhuofu Deng

**Affiliations:** College of Software, Northeastern University, Shenyang, Liaoning Province 110004, China

## Abstract

This paper proposes a novel lung nodule classification method for low-dose CT images. The method includes two stages. First, Local Difference Pattern (LDP) is proposed to encode the feature representation, which is extracted by comparing intensity difference along circular regions centered at the lung nodule. Then, the single-center classifier is trained based on LDP. Due to the diversity of feature distribution for different class, the training images are further clustered into multiple cores and the multicenter classifier is constructed. The two classifiers are combined to make the final decision. Experimental results on public dataset show the superior performance of LDP and the combined classifier.

## 1. Introduction

Lung cancer is among the most common medical conditions worldwide, and it is responsible for 1.56 million deaths as of the year 2012 [1]. Overall, 16.8% of people in the United States that are diagnosed with lung cancer survive for five years after the diagnosis, while its outcomes on average are worse in the developing countries [2]. It is showed that using low-dose computed tomography (CT) for early detection can significantly reduce the mortality of lung cancer [3]. Therefore, as a result, there is urgent desire for lung nodule CT image analysis in an efficient and convenient way.

Usually, a lung nodule is characterized by its bright appearance compared with its surrounding regions. Commonly, lung nodules can be classified into four different types according to their relative locations with neighbor pulmonary structures [4]. Here (A), (B), (C), and (D) are used to denote four types of lung nodule:

- Well-circumscribed nodule: without any connection to other pulmonary structures
- Juxtavascular nodule: with uncertain connections to surrounding vessels
- Pleural-tail nodule: with a thin connection between the nodule and the pleural
- Juxtapleural nodule: with a large proportional connection between the nodule and the pleural

**Figure 1:**
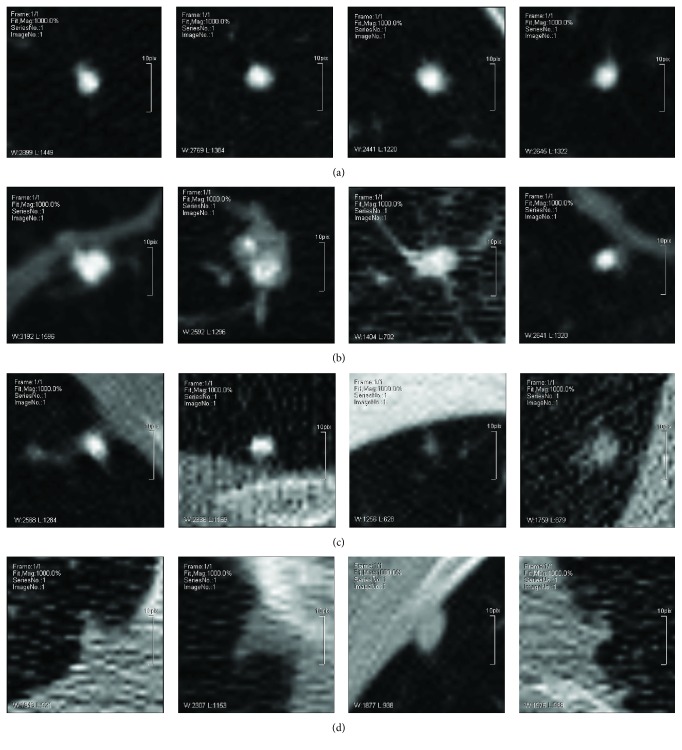
Sample images from the four types with (A), (B), (C), and (D) from left to right, respectively.

Demonstrations of four types of lung nodule images are shown in Figures 1(a)–1(d), respectively. The analysis of nodule morphology is a crucial step in the assessment of nodule malignancy [5]. Traditionally, this work is done by the expert manually. It is highly affected by his competence and status, and the efficiency is inevitably weakened for its time consuming. Therefore, automatic lung nodule type classification using computer vision technology is necessary to provide a supplementary medical treatment for the physician. The aim of this work is to automatically classify lung nodule CT image patches into four types with high performance.

Generally, medical image classification contains two main steps: (1) feature extraction and representation and (2) classifier construction. In the first stage, medical image is expressed with high dimensional feature vector, which denotes the texture, color, orientation, and so forth. In the second stage, supervised or unsupervised based learning methods are used to construct the classifier given the labeled training dataset. As a hot study area, there has been a lot of research on lung node image classification. Ciompi et al. focus on designing a descriptor which samples intensity profiles along circular patterns [5], and then a spectrum is computed by Fourier transform. The spectrum is clustered to form a library, and bag of frequency is used to construct the feature vector. Song et al. use the region-based energy method to label the background and foreground [6]. The locations of lung nodules with respect to the other structures are gained, and this information is used to construct the feature vector. Farag et al. first applied SIFT descriptor, and PCA and LDA are used for dimension reduction. Then, an adopted Daugman Iris Recognition algorithm is implemented and complex Gabor response is obtained [7]. Zhang et al. first used traditional supervised learning method to construct a bipartite graph [8]. The relationship between test image and training images is used to construct the ranking score and contribution score, and the final classification result is gained. Jacobs et al. propose a segmented-based method [9]. It characterizes the nodule as solid, part-solid, and nonsolid and then a supervised learning method is implemented. In another method, shape features such as smoothness and irregularity of a nodule are used to construct the feature representation [10]. Samala et al. use nine-feature descriptors for lung nodule representation which were often used by radiologists [11]. Lowe uses SIFT representation to characterize the feature of nodule, and then LDA is used to construct the classifier [12]. Maldonado et al. proposes a method that nodule patches are clustered to construct the feature dictionary, and then the testing nodule voxels are labeled [13]. Song et al. first clustered images to a sparse representation based on spectral analysis, and test image is formed with sparse representation. Finally, classifier is constructed by a fusing method [14]. Zhang et al. use a supervised learning method to find four probability values that belongs to each type [15]. Then, a weighed Clique Percolation method is implemented to discover the overlapping of lung nodules that belong to different type.

**Figure 2:**
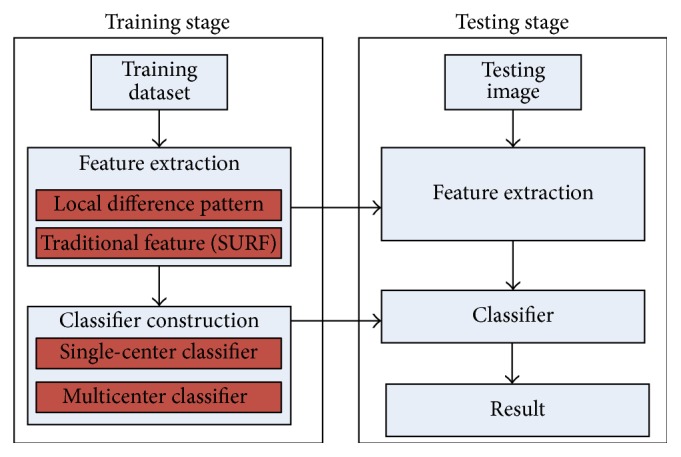
Framework of the proposed method.

There are many methods about lung nodule image classification. However, the complex structure of the medical image causes the classification high variance intraclass and high similarity interclass. Therefore, the automatic medical image classification is still a challenging problem. Most of the existing methods adopt generic feature representations which is commonly used in computer vision domain. These methods lack specialized analysis for the texture and shape of lung nodule. On the other hand, using one classifier scheme, whether supervised based or unsupervised based may not be well matched with the lung nodule classification. Facing the above mentioned problems, this paper proposes a novel lung nodule representation and image classification method. As shown in Figure 2, the training stage learns the classification model, and the model is used in testing stage for image classification. In feature extraction step, a novel feature Local Difference Pattern (LDP for abbreviation) is designed based on the gray level difference between lung nodule and its neighbors region. The LDP representation is more specialized and comprehensive. In the step of classifier construction, single-center classifier is first constructed using supervised learning and LDP feature representation. In the next step, labeled images are clustered into multiple centers using unsupervised learning method. The multicenter classifier is then constructed based on the basis of the similarity between the testing image and multiple centers. These two classifiers are combined to construct the final classifier. In testing stage, the image is represented as the same scheme in training stage, and the classification result can be gained using the final classifier. The main contributions of the paper are as follows:

- First, based on the analysis of the characteristic of lung nodule and the distribution of the corresponding tissues, a novel feature representation, LDP, is proposed. The new feature is suited for reflect the distinguishing feature of different types of lung nodule.
- Second, generative model and discriminative model are used to construct single-center and multicenter classifiers. These two classifiers complement each other, which makes the classification more robust.

The structure of this paper is organized as follows. Local Difference Pattern is given in Section 2. Classifier construction is given in Section 3. Experimental results are shown in Section 4.  Section 5 concludes this paper.

## 2. Local Difference Pattern

As shown in Figure 1, different types of lung nodule can be characterized by various features, while the size and gray level of the nodule itself could vary to a certain distance. So, this paper focuses on extracting the feature that reflects the gray level difference between the nodule and its neighbor regions.

**Figure 3:**
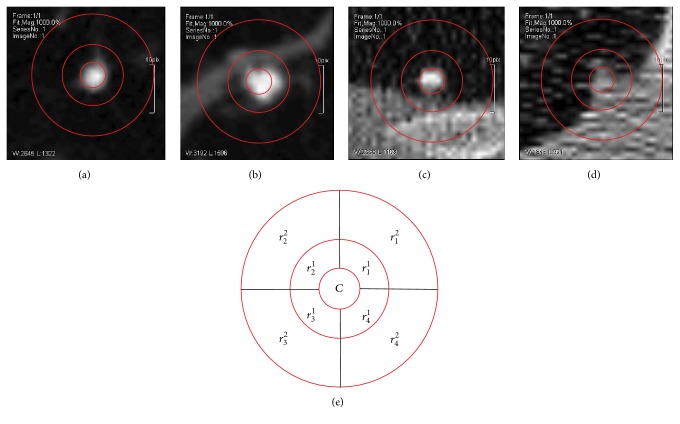
Demonstration of Local Difference Pattern. (a)–(d) are four types of lung nodule images, red circles denote the region used for feature extraction. (e) denotes the detail region partition used for feature extraction.

This paper proposes the Local Difference Pattern (LDP) to describe the local feature of lung nodule image. As shown in Figures 3(a)–3(d) give four types of lung nodule image, and each has three concentric circles with the nodule in the center circle. LDP is extracted according to the concentric circle regions.  Figure 3(e) gives the detailed information of subregion partition used for feature extraction. The center circle is denoted as* C*, and the out layer circles are divided into four parts according to four quadrants. *r*_*i*_^*j*^ is the average gray level of the corresponding region, where superscript* j* means the number of circle and subscript *i* means the number of quadrant.

**Figure 4:**
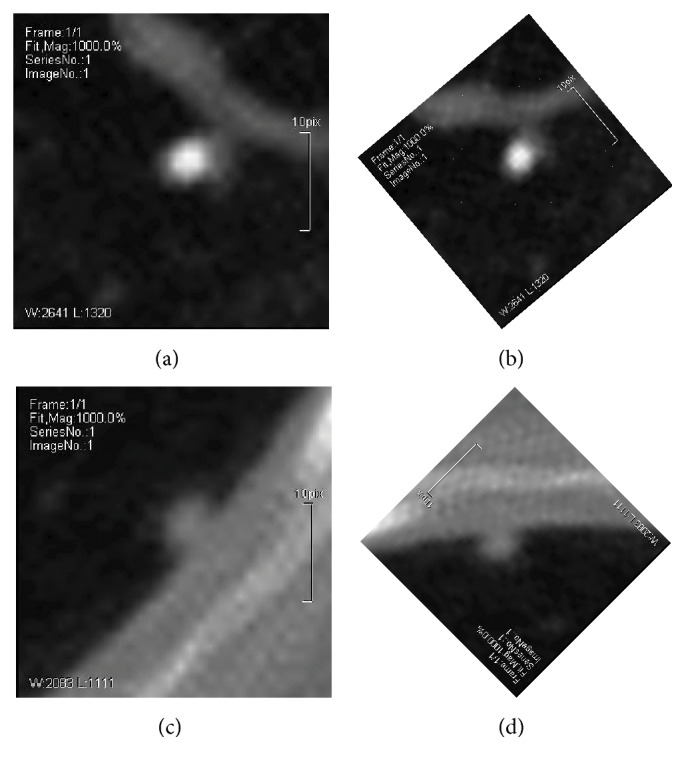
Rotation of the lung nodule images. (a) and (c) are the traditional images, while (b) and (d) are their rotated images according to the main direction, respectively.

Moreover, one of the most important objectives is the rotation invariant of the local feature. Before LDP extraction, some adjustment should be done to the original image patches. By the aid of design mode from other local feature, that is, SIFT, SURF, and so forth [16], the main direction of the lung nodule image is calculated first, and then LDP can be extracted in the rotated image according to the main direction, as shown in Figure 4. For the lung nodule images are collected with the same resolution, so the scale of the feature cannot be considered here.

In the light of the above description, LDP is defined as follows:

(1) LDPI=C,ri1,ri2,sign⁡ri1−C,sign⁡ri2−C,sign⁡ri1−ri+1 mod 41,sign⁡ri2−ri+1 mod 42,sign⁡ri1−ri2,1≤i≤4.

As shown in (1), LDP(*I*) means feature vector of lung nodule image *I*, which is composed of multidimensional data. sign⁡(*r*_*i*_^1^ − *C*) and sign⁡(*r*_*i*_^2^ − *C*) denote the gray level difference between the center and the outlier circles. sign⁡(*r*_*i*_^1^ − *r*_*i*_^2^) denotes the gray level difference between 1th and 2nd circle in different quadrant. sign⁡(*r*_*i*_^1^ − *r*_(*i*+1) mod 4_^1^) and sign⁡(*r*_*i*_^2^ − *r*_(*i* + 1) mod 4_^2^) denote the gray level difference between neighbor quadrants in a counterclockwise direction inside one concentric circle. Totally, a 29-dimensional feature vector is used to represent the LDP.

## 3. Classifier Construction

In this section, single-center classifier and multicenter classifier are constructed, respectively, and a combined one is further build. Illustrations are given in detail as follows.

### 3.1. Single-Center Classifier

Given the labeled image dataset, LDP feature is first extracted for each training lung nodule image, and then a supervised learning method is used straightly. Here, linear SVM is adopted to construct the classifier model, and it is called single-center classifier *f* ^*S*^. The classifier *f* ^*S*^ outputs the possibility that one image belongs to each type of lung nodule.

### 3.2. Multicenter Classifier

The lung nodule images are not easy to classify for there exist large intraclass variance and high interclass similarity. And due to the multiple distribution nature of diversity for the image, a single supervised classifier is probably insufficient to catch the diverse representations of one class data. Thus, this paper applies one more step to the algorithm. By implement clustering with 64-dimensional feature vector SURF of each training image, images with the same class label are further clustered to form some centers, which can be represented as follows:

(2) $$\begin{array}{c} C^{k}=\left\{\;C_{1}^{k},C_{2}^{k},\ldots ,C_{n}^{k}\;\right\},\;1\leq k\leq 4, \end{array}$$

where superscript *k* means the class label and subscript *i* denotes multiclusters in one class. *n* denotes the number of center. Given an image for testing, its probability that belongs to four types of lung nodules can be computed as follows:

(3) CFik=NumCikNumk,1≤k≤4,  1≤i≤5,

(4) S=CFi11+CFi22+CFi33+CFi44,

(5) fM=CFi11S,CFi22S,CFi33S,CFi44S.

As shown in (3), Num(*C*_*i*_^*k*^) denotes the number of training images in class *k* which belongs to *i*th center. Num(*k*) denotes the number of training images in class *k*. *CF*_*i*_^*k*^ denotes the frequency of center *i* in class *k*. Given a test image* X*, let *C*_*i*1_^1^, *C*_*i*2_^2^, *C*_*i*3_^3^, *C*_*i*4_^4^ be its centers of four lung nodule types; then, (4) and (5) can be used to construct the multicenter classifier *f* ^*M*^, which gives the values of probability that *X* belongs to each of four types, respectively:

(6) $$\begin{array}{c} F=w*f^{S}+\left(\;1-w\;\right)*f^{M}. \end{array}$$

As shown in (6), the single-center classifier and multicenter classifier are combined to get the final classifier *F*, where *w* is the weighted parameter.

## 4. Experimental Evaluation

### 4.1. Dataset and Program Implementation

In this section, the public available dataset is used for the experiment evaluation [17]. The dataset contains 379 lung nodule images with center position of nodule annotated, which are comprised of 50 distinct CT lung scans. The lung nodules are classified into four types according to the instruction by an expert.

The lung nodule images are cropped from the original CT images according to the position of nodule center. The original CT image is with a resolution of 512 pixel *∗* 512 pixel, and the cropped image patches are too small to implement the computer vision algorithm. Therefore, the cropped images are further interpolated to 160 pixel *∗* 160 pixel with the bicubic method. All the programs are implemented using Matlab 2012 programming language and tested on a Pentium Dual-2.4 CPU, 2 G RAM PC.

### 4.2. Parameter Setting

*L*_*r*1_, *L*_*r*2_, and *L*_*r*3_, denoted as the size of three concentric circles, along with classifier weight *w* and the number of multiclusters in each class *n* are evaluated with comprehensive testing. As shown in Table 1, the option range of *L*_*r*1_ is 20–45 pixels, with a step of 5 pixels, the option range of *L*_*r*2_ is 70–95 pixels, with a step of 5 pixels, the option range of *L*_*r*3_ is 100–125 pixels, with a step of 5 pixels, the option range of *w* is 0.3–0.8, with a step of 0.1, and the option range of *n* is 3–7, with a step of 1. So there are 6480 (6*∗*6*∗*6*∗*6*∗*5) combinations of parameters setting. After complete testing, *L*_*r*1_, *L*_*r*2_, and *L*_*r*3_, the radii of three concentric circles, are set with 35 pixels, 90 pixels, 105 pixels, respectively. The weight of combined classifier *w* is assigned with 0.6. The number of multiclusters in each class *n* is set as 5. This set of parameters gives the highest classification rate.

### 4.3. The Proportion of Training Dataset versus Classification Rate

The proportion of training dataset may have influence on classification rate of the algorithm. In this subsection, training dataset is selected randomly with the proportion from 10% to 90%, with a step of 5%, and the remainder is used for testing. The testing is performed many times and the average classification rate is computed.

**Figure 5:**
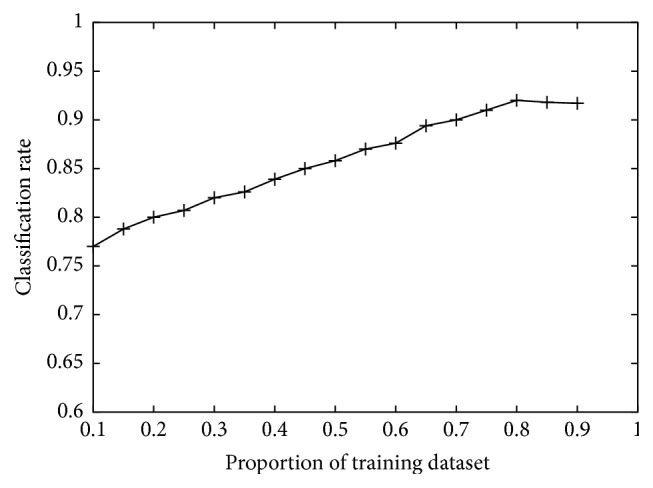
The influence of proportion of training dataset on classification rate.

Figure 5 gives the demonstration of proportion of training dataset versus classification rate. As can be seen from the figure, the classification rate is raised as the proportion of training dataset is increased. That means more training data can incorporate more information, and a better data representation diversity can be gained, and therefore the performance is enhanced. Meanwhile, when the proportion exceeds some value the classification rate is tend to be stable.

### 4.4. Average Classification Rate

**Figure 6:**
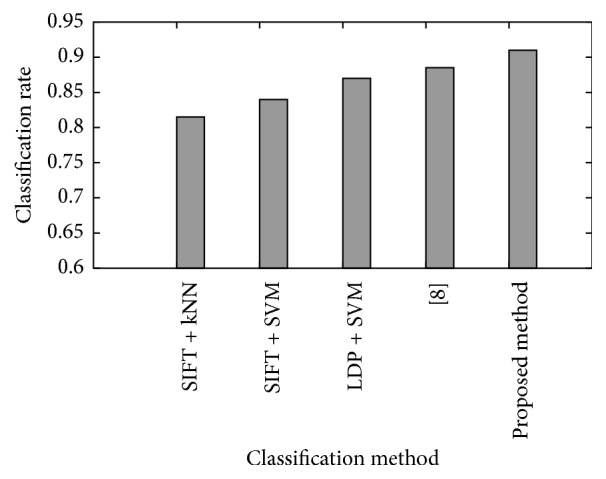
The classification rate among five methods.

In order to evaluate the classification rate comparison between different methods, five algorithms are used for testing, which are composed of various feature representation and classifier. The classification rate is the average value for different training dataset.  Figure 6 gives the comparison result. It is shown that LDP + SVM has a higher performance than SIFT + kNN and SIFT + SVM, which means LDP designed in this paper contains more useful information to represent the local feature. Among all five algorithms, the proposed method demonstrates the best performance.

### 4.5. ROC Testing

**Figure 7:**
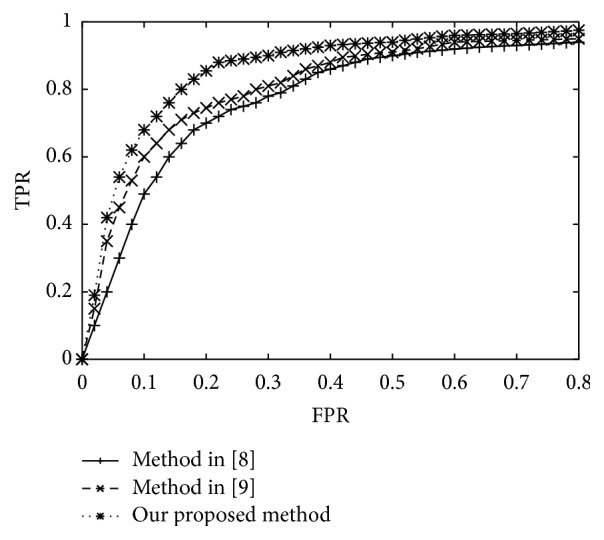
The ROC curve testing with different methods.

ROC curves are a regular tool for illustrating the performance of a classifier system, and the curve can be gained by plotting the true positive rate (TPR) against the false positive rate (FPR) at varied discrimination threshold settings. Some recent algorithms are chosen for comparison with our proposed one [8, 9], and the results are given in Figure 7. It can be seen clearly from the demonstration that the proposed method has the superior ROC curves characteristic.

## 5. Conclusion

This paper proposes a method for lung nodule image classification. First, a novel local feature representation, Local Difference Pattern, is designed, which can catch more information from the lung nodule and its neighbor regions. And a single-center classifier is constructed according to LDP and SVM. Then, a multicenter classifier is designed by clustering the SURF feature of lung nodule image and computing the similarity between testing image and multiple centers. Finally, the two classifiers are combined to implement the classification. The proposed method aims to extract more useful feature and decrease the gap between high variance intraclass and high similarity interclass. Evaluation on public dataset shows that our proposed method outperforms other methods for lung nodule image classification. Our future works will focus on designing more accurate feature representation methods for lung nodule image, such as autoencoder and convolutional neural network.

## Figures and Tables

**Table 1:** The values of parameters used in the proposed method.

Notation	Description	Option range	Determined value
*L*_*r*1_	Radius of 1st concentric circle	20–45 pixels (step with 5 pixels)	35 pixels
*L*_*r*2_	Radius of 2nd concentric circle	70–95 pixels (step with 5 pixels)	90 pixels
*L*_*r*3_	Radius of 3rd concentric circle	100–125 pixels (step with 5 pixels)	105 pixels
*w*	Weight of combined classifier	0.3–0.8 (step with 0.1)	0.6
*n*	Number of multiclusters	3–7 (step with 1)	5
